# Exceptional response to chemotherapy followed by concurrent radiotherapy and immunotherapy in a male with primary retroperitoneal serous Adenocarcinoma: a case report and literature review

**DOI:** 10.1186/s12885-019-5934-4

**Published:** 2019-07-30

**Authors:** Young Kwang Chae, Naira Saleem, Yoonhwan Roh, Haris Bilal, Pedro Viveiros, Bhoomika Sukhadia, Xiaoqi Lin, Muhammad Mubbashir Sheikh, Lee Chun Park

**Affiliations:** 1 Department of Medicine, Feinberg School of Medicine, Northwestern University, Chicago, IL- 60611 USA; 2 Department of Pathology, Feinberg School of Medicine, Northwestern University, Chicago, IL USA

**Keywords:** Primary retroperitoneal serous adenocarcinoma, PRSA, Chemotherapy, Radiotherapy, Nivolumab

## Abstract

**Background:**

Primary retroperitoneal serous adenocarcinoma (PRSA) is an extremely uncommon malignancy exclusively reported in females. Due to the rarity of the disease, it is difficult to establish a standardized treatment.

**Case presentation:**

We describe a unique case of PRSA in a 71-year-old male who presented with right-sided lower back pain and numbness. Magnetic resonance imaging identified a mass invading the adjacent psoas muscle and twelfth rib. Tissue biopsy confirmed poorly differentiated PRSA. Patient was initially treated with neoadjuvant carboplatin and paclitaxel chemotherapy regimen. This resulted in complete radiological resolution of the tumor. However, 12 weeks later, rapid recurrence was noted on follow-up CT scan. The patient was then treated with external radiotherapy with concurrent nivolumab, an anti-PD-1 antibody. The patient displayed a positive response to treatment with reduction in primary tumor and metastases and had a sustained disease control.

**Conclusion:**

Treatment with radiotherapy in combination with anti-PD-1 antibody could be an effective modality of management for PRSA.

**Electronic supplementary material:**

The online version of this article (10.1186/s12885-019-5934-4) contains supplementary material, which is available to authorized users.

## Background

Primary retroperitoneal serous adenocarcinoma (PRSA) is a rare clinical entity. To date, a handful of cases have been reported all of which occurring in female patients [1–8]. While there have been some reports of mucinous subtype of retroperitoneal adenocarcinoma in males [9, 10], no case of serous subtype has been presented in this subgroup to the best of our knowledge. The origin of this neoplasm is still unclear. Several hypothesis such as celomic metaplasia, cystic endosalpingiosis, and endometriosis have been proposed [3–5, 7, 11–14]. Due to scarcity of typical cases and comparable biological behavior with ovarian serous carcinoma, the most commonly reported treatment for PRSA is surgical resection of the mass with adjuvant platinum based chemotherapy [4, 5, 14–16]. We describe a unique case of PRSA in a male, who was treated with carboplatin/paclitaxel therapy and subsequently had a complete radiological response. However, there was rapid recurrence of the tumor. The patient was then subjected to combination treatment with radiotherapy (RT) and immunotherapy and exhibited a favorable response with reduction in primary tumor size and metastases.

## Case presentation

**Fig. 1:**
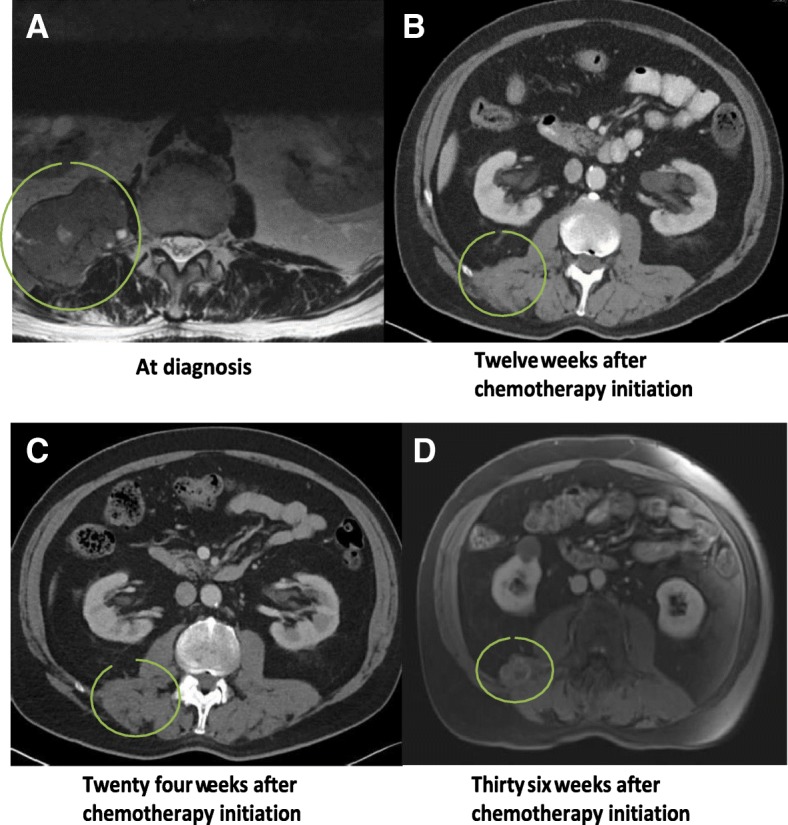
Imaging studies demonstrating the course of tumor response to chemotherapy. **a** Retroperitoneal mass at time of diagnosis. **b** Reduction in tumor size after 12 weeks of therapy such that it is difficult to delineate from surrounding soft tissue. **c** Complete resolution of mass with minimal fat stranding **d** Recurrence of multinodular cystic mass at the site of origin. Circles highlight the location of the neoplasm

A 71-year-old male presented to the clinic with lower back pain and numbness on the right side in January 2017. The magnetic resonance imaging (MRI) of the lumbar spine showed a mass in the right retroperitoneum (Fig. 1a). He had been previously treated for localized prostate adenocarcinoma (Gleason stage 3 + 3 = 6) with brachytherapy 9 years ago and has been in remission ever since. Since the patient was taking rivaroxaban for atrial fibrillation, there was high suspicion for primary retroperitoneal hematoma. However, computed tomography (CT) scan of the abdomen revealed a right retroperitoneal mass associated with retroperitoneal lymphadenopathy, thus favoring the diagnosis of malignancy. MRI of the abdomen and pelvis outlined a lobulated T1 isointense right pelvic mass measuring 9.1 × 5.3 × 14.0 cm invading the adjacent psoas muscle, diaphragm, and right pleural space with encroachment onto the posterior right twelfth rib. Many sub-centimetric T2 hyperintense lesions in the posterior left iliac bone were noted, raising the suspicion for metastatic disease. Enlarged retroperitoneal and retrocrural lymph nodes were also seen. Scrotal ultrasound did not show any testicular mass. The imaging studies did not depict any other lesion that could be deemed as the primary source of neoplasm.

**Fig. 2:**
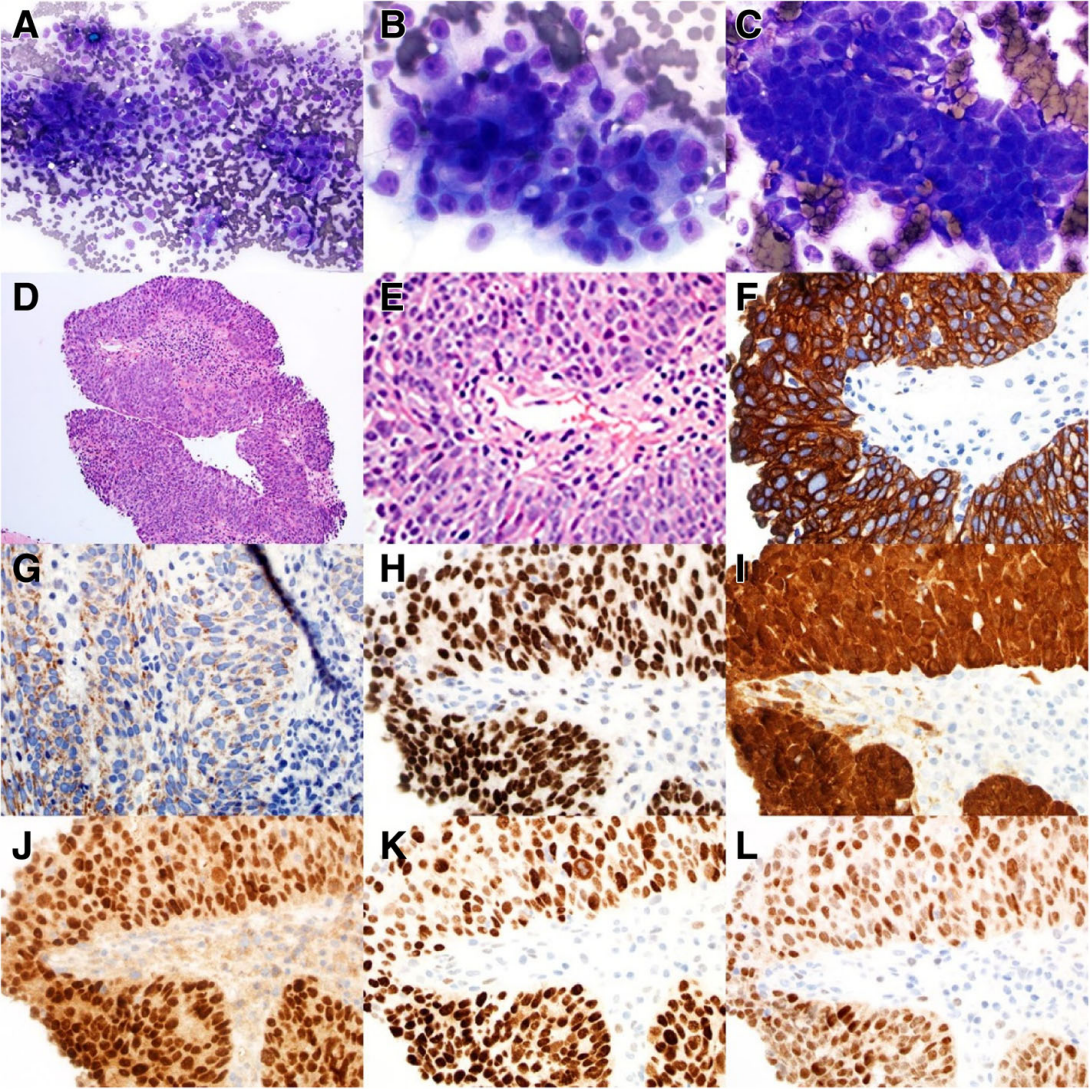
Cytomorphology, histology and immunochemistry of primary peritoneal high grade serous carcinoma. **a** to **c**: Diff-Quick stain of touch preparation of cores, 20x, 60x and 60x, respectively; **d** and **e**: Hematoxylin and eosin stain of core biopsies, 10x and 400x, respectively; **f** to **l**: Immunohistochemical stains of the core biopsy. CK7 (1**f**), calretinin (1**g**), WT-1 (1**h**), p16 (1**i**), PAX-8 (1**j**), p53 (1 **k**) and ER (1**l**), 40x

Next, CT-guided biopsy of the mass was performed which revealed a high-grade poorly differentiated adenocarcinoma, serous sub-type. Immunohistochemistry was positive for WT1, PAX-8, p16, p53, ER, BerEP4, focally positive for calretinin and CK 5/6. Thus, the diagnosis of PRSA was made after a thorough work up (Fig. 2). PD-L1 status was positive, PD-L1 present in 10% of tumoral cells and 30% of tumor infiltrating immune cells.

The analysis of tissue DNA by Tempus (Tempus biotechnology, Chicago, IL) showed two potentially actionable mutations: *NF1* and *TP53*. Both mutations were confirmed by circulating tumor DNA analysis by Guardant 360 (Guardant Health, Redwood city, CA), and described as somatic alterations, with a third somatic mutation, *FGFR1,* identified. However, there was no plausible therapeutic strategy for PRSA that could target these mutations. Table 1 summarizes the results of tissue DNA analysis. A total of eight mutations were detected. The tumor mutational burden was reported to be 1.37 non-synonymous mutations per/Mb. No mutations on mismatch repair genes were encountered.

**Table 1:** Result of tissue DNA analysis by TEMPUS (TEMPUS biotechnology, Chicago, IL) showing potentially actionable mutations and variants of unknown significance

Somatic variants with potentially actionable mutations	Mechanism	Allelic fraction (%)	Mutation effect
TPF3	Point Mutation	47.91	p.G245D
NF1	Point Mutation	46.44	p.Q209
Variants of unknown significance (VUS)			
NCOR2	Point Mutation	30.42	p.D1708G
CARD10	Point Mutation	27.17	p.R424W
FUS	Splice Site	7.05	c.1542-7_1 542-5delTTT
ADAMTS20	Point Mutation	3.59	p.Y948fs
BRPF3	Point Mutation	3.37	p.P854fs
EPHA3	Point Mutation	29.94	p.L588 V

Additional evaluation with brain MRI identified an asymptomatic 3 mm focal enhancement in the left lateral frontal lobe cortex. Since there was no mass effect, possibility of a metastatic lesion seemed unlikely. Nuclear bone scan highlighted diffusely increased uptake within the right twelfth rib, corresponding to the area where the rib was seen to be involved by the adjacent malignant tissue on prior CT and MRI scans.

On the basis of limited information available in the existing medical literature, the most commonly used therapy for PRSA has been surgical resection of the tumor with adjuvant platinum-based chemotherapy, similar to treatment of ovarian serous adenocarcinoma. Hence, the patient was treated with neo-adjuvant carboplatin and paclitaxel combination (Carboplatin AUC 5 every 3 weeks and paclitaxel 80 mg/m2 intravenously every week) for six cycles. Subsequent imaging after 12 weeks demonstrated a reduction in the size of the malignant mass and lymph nodes (Fig. 1b, c). The tumor response map illustrated the gradual decrease in somatic mutation burden from 1.7 to 0.4% over the course of treatment (Additional file 1: Figure S1).

**Fig. 3:**
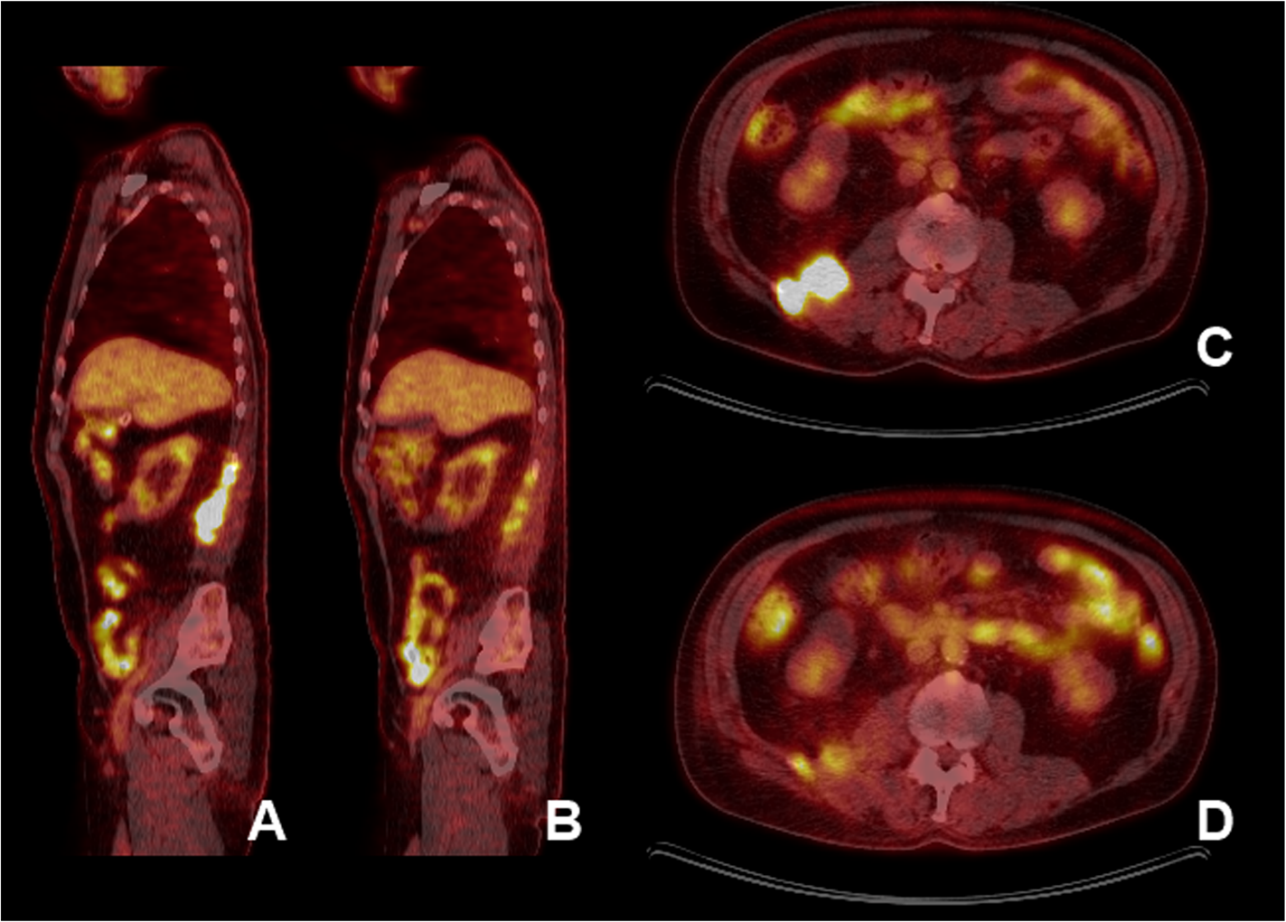
PET CT scan images showing changes in metabolic activity at the site of recurrent tumor after starting treatment with concurrent radiotherapy and immunotherapy. (**a**) and (**c**) illustrate a high standardized uptake values (SUV) in sagittal and axial sections respectively. It decreased in next 12 weeks following therapy as indicated by relatively decreased metabolic activity of tumor in sagittal (**b**) and axial sections (**d**)

Six months after starting treatment, follow-up CT scan depicted an essentially resolved retroperitoneal mass with normal-sized retroperitoneal and retrocrural lymph nodes. Despite a dramatic initial response, the malignant mass re-emerged 3 months later on the follow-up CT scan. On MRI, it appeared as a multi-lobulated cystic mass, consisting of three representative nodules measuring 2.4 × 3.0 cm, 1.7 × 2.3 cm and 1.0 × 1.1 cm (Fig. 1d) and extending from the level of T12 to L4/L5 vertebrae. No new enlarged lymph nodes, metastatic bone or brain lesions were identified. The baseline positron emission tomography (PET) scan was consistent with description of the MRI (Fig. 3a, c). In addition, there were several areas of increased metabolic uptake in upper abdominal retroperitoneal lymph nodes as well as left supraclavicular/anterior mediastinal lymph nodes consistent with metastases.

Given the extensive and aggressive nature of the disease with no plausible therapeutic strategy for the identified mutations, off-label treatment with concurrent external RT and immunotherapy was started. Immunotherapy is believed to enhance the immunogenic effect of RT against malignant cells [17]. Patient received radiation to retroperitoneum and left side of the neck with a dose of 39 Gy divided in 13 daily fractions. Alongside, he was given anti-programmed cell death protein-1 (anti-PD-1) antibody nivolumab (240 mg fixed intravenous dose given once every 2 weeks). Following treatment for 3 months, PET-CT revealed a significant reduction in the size of the lymph nodes in the left supraclavicular region, upper abdomen and right posterior retroperitoneum (Fig. 3b, d). However, new sites of hypermetabolic activity were noted in mediastinal lymph nodes which reduced in size on a later follow-up.

**Fig. 4:**
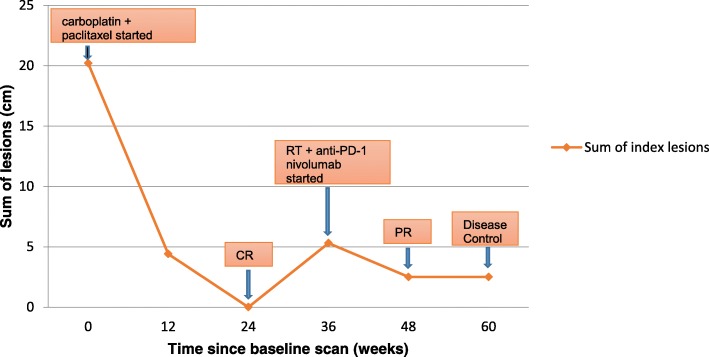
Changes in tumor burden under chemotherapy followed by radiotherapy and immunotherapy combination. RT: Radiotherapy, PDL1: programmed death ligand 1, CR: complete response, PR: partial response

Figure 4 illustrates the changes in tumor burden during the entire course of treatment. Tumor burden and response to therapy were evaluated using the revised Response Evaluation Criteria in Solid Tumors (RECIST) 1.1 [18].

Patient has been following up for 15 months since the initial diagnosis. He is currently tolerating treatment with nivolumab with negligible side effects and good performance status (Zubrod score 0). The plan is to continue nivolumab with imaging studies at every 12-week interval. Written consent was obtained from the patient for case publication.

## Discussion

Primary retroperitoneal tumors account for 0.2–0.3% of all tumors. The common histological patterns include fibrosarcoma, lymphoma and teratoma. [8] The epithelial subtype is the least prevalent of all. PRSA is an epithelial tumor bearing histological resemblance with ovarian serous carcinoma. To date, there have been eight reported cases of PRSA, all of which were in females [1–8]. We describe the first case of PRSA in a male.

Primary retroperitoneal mucinous cystadenocarcinoma (PRMC) is one of the subtypes of retroperitoneal neoplasm which also resembles the ovarian mucinous tumors. It is also a rare entity with less than sixty cases reported in current literature [9]. Moreover, similar to PRSA, PRMC is less prevalent in males [10]. There has been a unique report of a primary retroperitoneal mullerian adenocarcinoma composed of mixed epithelial components: papillary, serous, mucinous and endometrioid [15]. However, the biological explanation for a greater propensity of developing non-serous neoplasms compared to their serous counterparts is still unclear.

Some of the proposed theories to explain pathogenesis of PRSA and PRMC include neoplastic alterations in the metaplastic celomic epithelium, extra-ovarian endometriosis [5, 11], cystic endosalpingiosis [5, 14], and heterotopic ovarian tissue [3, 4, 6, 11, 16]. Metaplasia of celomic epithelium leading to the development of PRSA is the most widely accepted theory [1, 3–5, 7, 12, 13]. One study demonstrated that a small portion of benign mucosal epithelium adjacent to the tumor mass had differentiated into tubal morphology providing a solid evidence in support of this theory [7]. The mullerian origin of these tumors is advocated by synchronous occurrence of these malignancies in tissues embryologically derived from the mullerian tube [5]. Primary peritoneal carcinoma may arise as synchronous primary tumors from different foci of mullerian epithelium scattered within the peritoneal lining [19–21]. Since our case is in a male patient, the hypotheses of heterotopic ovarian tissue and mullerian tube origin seem unlikely.

In light of the striking resemblance in biological and therapeutic behavior between primary retroperitoneal epithelial cancers and ovarian epithelial cancers [1, 3], both have been managed in a similar fashion. The *en bloc* surgical resection of malignant mass with sufficient tumor-free margins remains the mainstay of treatment given the locally invasive tendency of retroperitoneal epithelial cancer [4, 5, 14–16]. It has been advocated that adjuvant chemotherapy with carboplatin alongside docetaxel or paclitaxel be given following surgery in patients with risk factors for recurrence [4]. Although surgical resection of tumor with adjuvant chemotherapy is accepted as an appropriate treatment for PRSA [6–8], definitive guidelines cannot be established.

Based on the aforementioned facts, our patient was treated initially with chemotherapy consisting of carboplatin and paclitaxel with an intent to shrink the tumor to a surgically resectable size. The patient exhibited a complete radiological response after six cycles. Out of the eight cases previously described in literature, one patient received upfront chemotherapy with carboplatin and cyclophosphamide as the first line treatment and underwent a partial response [3]. Two patients underwent surgical resection followed by chemotherapy and were in remission after 6 and 21 months of primary treatment, respectively [6, 8]. However, in three other cases, no response was shown; these patients were then subjected to additional chemotherapy but none of them exhibited complete response [4, 5, 7]. Therefore, this is the only case to the best of our knowledge that presented a complete radiological response with chemotherapy as first line treatment. Nonetheless, the tumor recurred within 3 months of therapy, reflecting the aggressive nature of PRSA [15].

RT affects dead or dying cells that release antigens like calreticulin and high-mobility group protein B1 that activates dendritic cells, which in turn activate the antigen-specific T-cells to mount tumor specific immune response. The surviving irradiated tumor cells display increased expression of death receptor Fas, intercellular adhesion molecule (ICAM-1) and major histocompatibility complex 1 that allows enhanced recognition by activated T-cells. In addition, radiation therapy also upregulates the expression of T-cell inhibitory proteins such as PD-1 and CTLA-4 on tumor cells that suppress the host immunity [22]. Several drugs that target these immune checkpoints have been developed. These drugs may augment the immunostimulatory effects of RT. Thus, there is growing evidence suggesting a synergistic role of radiation therapy with immunotherapy for treatment of several malignancies. In one study, combination of RT with ipilimumab (an anti-CTLA-4 antibody) showed an increased median overall survival (19 months vs. 10 months, *p* = 0.01) and complete response rate (25.7% vs. 6.5%, *p* = 0.04) when compared to ipilimumab alone [23]. Similarly, RT concurrent with anti-PD-1 agents (pembrolizumab or nivolumab) has depicted better overall survival rates, good tolerability and higher tumor response rates in metastatic melanoma when compared with single agent treatment [24]. Thus, preliminary observations suggest that synergy between radiation and immunotherapy could be an effective therapy in advanced cancer patients.

Another interesting aspect of this case was the estrogen receptor (ER) positivity. ER expression may suggest benefit of adding an anti-estrogenic agent or aromatase inhibitor to the treatment regimen and could be attempted as a next-line therapy. However, there is only one prior case study that reported ER positive PRSA, but no anti-estrogenic therapy was used [5].

## Conclusion

We report a unique case of primary retroperitoneal serous adenocarcinoma in a 71-year-old male. A complete radiological response to upfront platinum-based chemotherapy as observed in our patient has not been previously described. However, following rapid recurrence, institution of immunotherapy-radiotherapy combination led to decrease in tumor size followed by durable disease control. This suggests immunotherapy and RT synergy could be further explored as a potential treatment option for the management of PRSA.

## Additional file

Additional file 1: **Figure S1.** Tumor response map illustrating more than 50% decrease in somatic alteration burden following chemotherapy. (DOCX 61 kb)

## Data Availability

All data and materials used are not available publicly to protect patient identity but could be available from the corresponding author on special request.

## Abbreviations

Anti-PD-1: Anti programmed cell death protein-1
CT: Computed tomography
MRI: Magnetic resonance imaging
PET: Positron emission tomography
PRMC: Primary retroperitoneal mucinous adenocarcinoma
PRSA: Primary retroperitoneal serous adenocarcinoma
RT: Radiotherapy
