# Supplementary figures and images for: Exceptional response to chemotherapy followed by concurrent radiotherapy and immunotherapy in a male with primary retroperitoneal serous Adenocarcinoma: a case report and literature review
Source: BMC Cancer. 2019 Jul 30;19:748. doi: 10.1186/s12885-019-5934-4 (PMC6668104; doi:10.1186/s12885-019-5934-4)

0.4%

1.7%


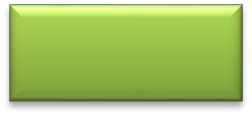


Somatic Alteration Burden

#
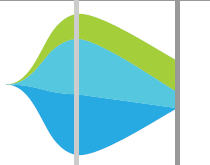


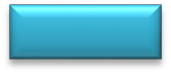


24 weeks after baseline


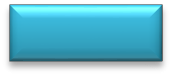


Baseline

Supplement: Supplementary file 1 — Figure S1. Tumor response map illustrating more than 50% decrease in somatic alteration burden following chemotherapy. (DOCX 61 kb) [file 12885_2019_5934_MOESM1_ESM.docx]
